# Determinants of poor adherence to secondary antibiotic prophylaxis for rheumatic fever recurrence on Lifou, New Caledonia: a retrospective cohort study

**DOI:** 10.1186/1471-2458-13-131

**Published:** 2013-02-12

**Authors:** Brunelle Gasse, Noémie Baroux, Bernard Rouchon, Jean-Michel Meunier, Isabelle De Frémicourt, Eric D’Ortenzio

**Affiliations:** 1 Centre médical de Wé, Circonscription médico-sociale de Drehu, Direction de l’Action Communautaire et de l’Action Sanitaire de la Province des Iles, Nouméa, Nouvelle-Calédonie; 2 Institut Pasteur de Nouvelle-Calédonie, Réseau International des Instituts Pasteur, Unité d’Epidémiologie des Maladies Infectieuses, Nouméa, Nouvelle-Calédonie; 3 Agence Sanitaire et Sociale de la Nouvelle-Calédonie, Cellule du Rhumatisme Articulaire Aigu, Nouméa, Nouvelle-Calédonie; 4 Cabinet de Cardiologie, Nouméa, Nouvelle-Calédonie; 5 Direction de l’Action Communautaire et de l’Action Sanitaire de la Province des Iles, Nouméa, Nouvelle-Calédonie

**Keywords:** Acute rheumatic fever, Rheumatic heart disease, Patient compliance, Antibiotic prophylaxis, Melanesia, New Caledonia

## Abstract

**Background:**

Incidence of acute rheumatic fever (ARF) and prevalence of rheumatic heart disease (RHD) in the Pacific region, including New Caledonia, are amongst the highest in the world. The main priority of long-term management of ARF or RHD is to ensure secondary prophylaxis is adhered to. The objectives of this study were to evaluate rates of adherence in people receiving antibiotic prophylaxis by intramuscular injections of penicillin in Lifou and to determine the factors associated with a poor adherence in this population.

**Methods:**

We conducted a retrospective cohort study and we included 70 patients receiving injections of antibiotic prophylaxis to prevent ARF recurrence on the island of Lifou. Patients were classified as “good-adherent” when the rate of adherence was ≥80% of the expected injections and as “poor-adherent” when it was <80%. Statistical analysis to identify factors associated with adherence was performed using a multivariate logistic regression model.

**Results:**

Our study showed that 46% of patients from Lifou receiving antibiotic prophylaxis for ARF or RHD had a rate of adherence <80% and were therefore at high risk of recurrence of ARF. Three independent factors were protective against poor adherence: a household with more than five people (odds ratio, 0.25; 95% confidence interval [CI], 0.08 to 0.75), a previous medical history of symptomatic ARF (odds ratio, 0.20; 95% CI, 0.04 to 0.98) and an adequate healthcare coverage (odds ratio, 0.21; 95% CI 0.06 to 0.72).

**Conclusions:**

To improve adherence to secondary prophylaxis in Lifou, we therefore propose the following recommendations arising from the results of this study: i) identifying patients receiving antibiotic prophylaxis without medical history of ARF to strengthen their therapeutic education and ii) improving the medical coverage in patients with ARF or RHD. We also recommend that the nurse designated for the ARF prevention program in Lifou coordinate an active recall system based on an updated local register. But the key point to improve adherence among Melanesian patients is probably to give appropriate information regarding the disease and the treatment, taking into account the Melanesian perceptions of the disease.

## Background

Acute rheumatic fever (ARF) and rheumatic heart disease (RHD) represent the first cause of cardiac mortality among children and young people in developing countries [1]. The worldwide prevalence of RHD is estimated at 15.6 million people, with an annual incidence of ARF of 470,000. The burden of mortality still concerns 230,000 people per annum, caused by infective endocarditis and heart failures [2].

Since the 1980s, recommendations of the World Health Organization (WHO) promote secondary prevention as the cornerstone of control programs [3]. Secondary prevention of rheumatic fever is defined as the continuous administration of specific antibiotics to patients with a previous attack of rheumatic fever, or well-documented rheumatic heart disease. The purpose is to prevent colonization or infection of the upper respiratory tract with group A beta-hemolytic streptococci and the development of recurrent attacks of rheumatic fever [4]. Secondary prevention is considered to be the most cost-effective strategy to reduce mortality and morbidity [4]. Based on local and central registers, its objectives are to identify new cases of ARF and RHD, standardize and improve the diagnosis, management and follow-up of the patients, train health agents and communities to ARF, and maximize the administration of secondary prophylaxis [5,6]. The recommended secondary prophylaxis consists of intramuscular injections of benzathine benzylpenicillin G (BPG), every three to four weeks, the dosage being adapted to patient’s weight [7]. Patients with proven penicillin allergy should be managed with twice-daily oral erythromycin [4]. Recommendations on the frequency of intramuscular injections and the duration of secondary prophylaxis vary between authorities. The WHO does not specify whether injections should be administered every 3 weeks or every 4 weeks. The appropriate duration of secondary prophylaxis is determined by a number of factors, including age, time since the last episode of ARF, ongoing risk of streptococcal infections and potential harm from recurrent ARF [4,8,9]. Its effectiveness in reducing rates of streptococcal pharyngitis, recurrences of ARF and the progression of RHD is clearly assessed, and might even lead to a regression of mild to moderate valvular lesions within a decade [10-12].

Nevertheless, poor adherence to treatment is the main impediment to secondary prevention. Defined as the concordance between the patient’s behavior and the care provider’s recommendations [13], adherence is a rate that can range from 0 to more than 100% [14]. Global adherence to treatment of chronic diseases in developed countries averages only 50%, particularly affecting the poor population. As many factors interact and interfere with adherence, it is considered as a multidimensional phenomenon [15].

Multiple measures of adherence to secondary prophylaxis of ARF were assessed since the 1970s, using various tools, such as the annual rate of BPG injections, the percentage of positive-for-penicillin urines, or the time interval and attendance at visiting specialist and echocardiographic appointments, but only few studies aimed at determining the factors associated to adherence [16-21]. Even though the level of adherence required to prevent further episodes of ARF is not known, the objective is to reach 100% of the annual expected BPG injections, with a recommended benchmark of 80% [9]. Patients receiving less than 80% of prescribed doses are considered at high risk of recurrence of ARF [6].

It is in the Pacific region that the highest prevalences of RHD in the world are found [22]. In New Caledonia, the prevalence of RHD was estimated at 9/1,000 in school-children in their fourth year of primary school [23] but there are no data relating to the level of adherence. The objectives of this study were to evaluate rates of adherence in people receiving BPG injections prophylaxis in Lifou and to determine the factors associated with a poor adherence in this population.

## Methods

### Ethics statement

Ethical clearance was obtained from the Public Health and Social Agency of New Caledonia (Agence Sanitaire et Sociale de la Nouvelle-Calédonie). The study was also approved by the French consultative committee for the data processing in health research (Comité Consultatif sur le Traitement de l'Information en matière de Recherche dans le domaine de la Santé, CCTIRS). Informed written consent was obtained from adults or from the child’s parents before conducting the interviews and after reading them information about the study. All data analyzed were confidential.

### Setting

The study took place in the tropical island of Lifou, located 100 km from New Caledonia’s main island, a French territory in the South Pacific. With a surface of 1,200 km^2^ and a population of 8,627 inhabitants (Census 2009, New Caledonian Institute for Statistics and Economics, ISEE), Lifou is divided into three districts and 37 tribes. Population is comprised of 96% of Melanesian whose income comes mainly from agriculture. According to ISEE 52% of the households are considered poor by the local criteria (Monthly household income < 75,000 XPF = 800 USD). Dwellings can be concrete, semi-concrete or traditional huts, mostly water and electricity-supplied. The main religion is Protestantism.The health care system includes two public health centers (We and Xepenehe), two private general practitioners (GPs) and a few private nurses. There is a nurse designated for the ARF prevention program, in Lifou acting as a link between the health agency in charge of the ARF prevention program based in the main island and local health professionals. There are no cardiologists or radiologists. Patients sometimes need to travel to the main island of New Caledonia by plane or ferry to attend the recommended annual echocardiography. Social security coverage is provided if the RHD is declared to the social welfare system. Patient with an adequate healthcare coverage do not have to advance medical expenses for the access to a cardiologist for regular follow up visits. . Otherwise, a copayment is required. Administration of BPG injections is free of charge, regardless of the patient’s medical coverage.

### Study design

We conducted a retrospective cohort study in Lifou and we included all patients receiving BPG injections prophylaxis over at least a period of 12 consecutive months, from the 1^st^ of January 2011 to the 31^st^ of December 2011.

The WHO adherence project has adopted the following definition of adherence to long-term therapy: « the extent to which a person's behaviour - taking medication, following a diet, and/or executing lifestyle changes, corresponds with agreed recommendations from a health care provider [15]. In our study, the rate of adherence was obtained by dividing the number of injections of BPG administered by the number of the expected injections in 2011 (n = 17), considering a frequency of 3 weeks. This 3 weekly frequency is the frequency recommended by the Caledonian health agency in charge of the ARF prevention program at the time of this study. Patients were then classified as “poor-adherent” when the rate of adherence was <80% of the expected injections and as “good-adherent” when it was ≥80% [6,9].

### Data collection

Data were collected from patients’ medical records and through a standardized questionnaire administered face to face by a single interviewer (BG). The questionnaire went through a pilot phase for 10 persons. For very young children, one of the parents at least was interviewed. For children aged less than 16 years old and able to understand the questions, we interviewed the child and one of his parents at least. We collected information about demographic and socio-economic characteristics, health care team and system-related factors, condition-related factors, therapy-related factors and patient related-factors, according to WHO recommendations [24]. All questions were close-ended with binary choices. To prevent information bias, we limited questions (n = 3) referring to patient recall (minimal information on ARF/RHD, reason for not taking the medication, hospitalization at diagnosis).

### Statistical analysis

Statistical analysis was performed by using Stata Statistical Software Release 11 (StataCorp. 2009, College Station, TX: StataCorp LP). Continuous variables were summarized using means ± standard deviation or median and interquartile range (IQR). Frequencies of potential factors between good-adherent and poor-adherent patients were compared by Fisher’s exact test. All statistical tests were two-tailed, and p values of less than 0.05 were considered to indicate statistical significance. We used logistic regression to examine the association between potential factors and the likelihood of a favorable outcome. Odds ratios (OR) and 95 percent confidence intervals (CI) were used to quantify the strength of these associations. Variables with a two-sided p value <0.25 were introduced in the multivariable logistic regression model. Multiplicative interactions were tested for their significance at the 0.05 level. A backward stepwise method was used to develop an optimal multivariable logistic model. Stata software includes automatic checks for collinearity. The Hosmer-Lemeshow goodness-of-fit test was used to evaluate the final regression model.

## Results

### Baseline characteristics of the population study

**Figure 1:**
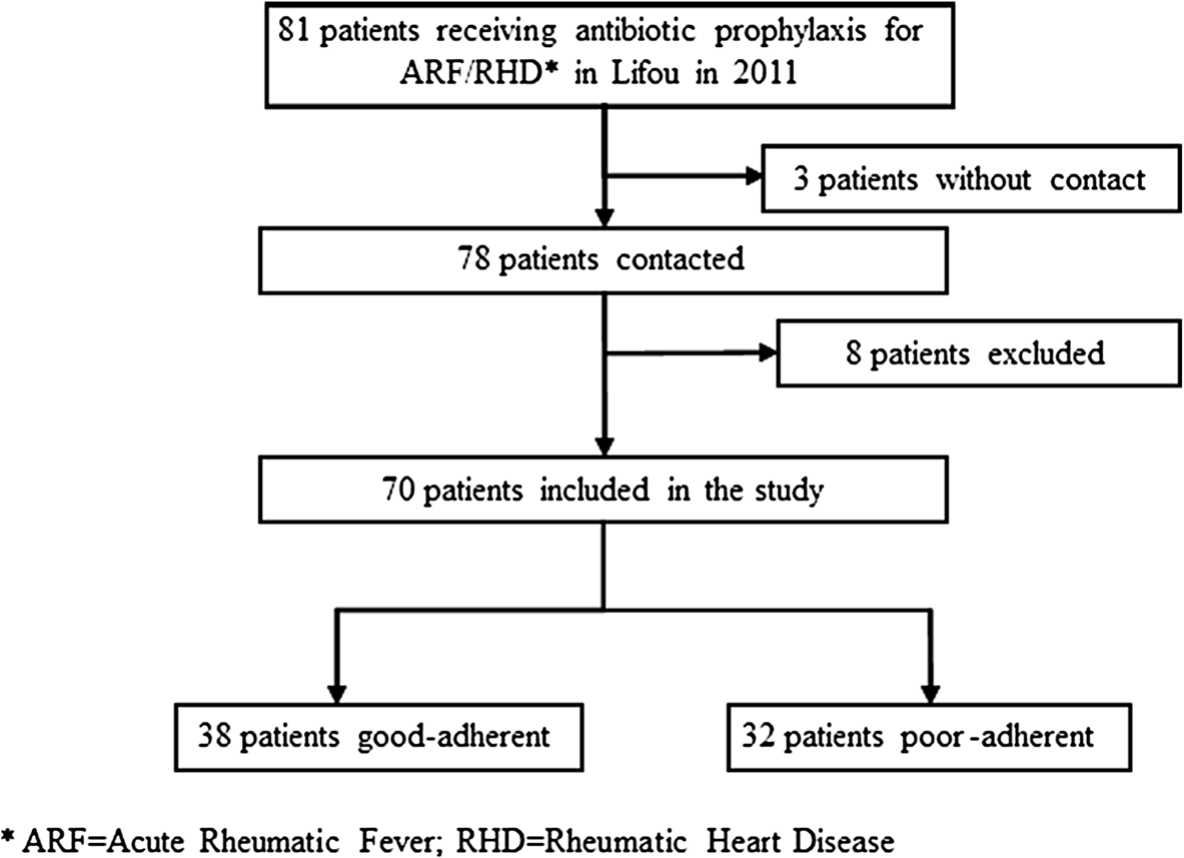
Flow chart of the enrollment of subjects, Lifou, New Caledonia, 2011.

A total of 81 persons with a history of ARF or RHD and taking antibiotic prophylaxis in 2011 were identified in Lifou, given a prevalence of 9.4/1,000 inhabitants. Among them, 11 were excluded: three were unreachable, three took oral antibiotic prophylaxis and five started antibiotic in the year 2011 (Figure 1). In the end, 70 patients were included in the study and the participation rate was 100%. The mean age of our population sample was 22.3 ± 11.6 years, 43% were children aged less than 16 years old and 63% were female. Baseline characteristics of our population are presented in Table 1.

**Table 1:** Characteristics of the 70 patients receiving antibiotic prophylaxis to prevent acute rheumatic fever recurrence, Lifou, New Caledonia, 2011

**Characteristics**	***no./total no.***	**%**
***Rate of adherence***		
<50%	12/70	(17)
50%-79%	20/70	(29)
≥80%	38/70	(54)
***Age *– *year***		
*Median (Interquartile Range)*	17	(13 – 32)
***>16 years old***		
No	40/70	(57)
Yes	30/70	(43)
***Sex***		
Female	44/70	(63)
Male	26/70	(37)
***Ethnicity***		
Mixed-origin*	4/70	(6)
Melanesian	66/70	(94)
***Disctrict of residence***		
Wetr	41/70	(59)
Gaïcha	10/70	(14)
Lossi	19/70	(27)
***Household income***		
≥150,000 XPF (1,600 USD) / month	43/67	(64)
<150,000 XPF (1,600 USD) / month	24/67	(36)
***Religion***		
Catholic	12/70	(17)
Protestant	49/70	(70)
Other	9/70	(13)
***History of acute rheumatic fever***		
No	13/70	(19)
Yes	57/70	(81)
***Diagnosis of rheumatic heart disease***		
No	16/70	(23)
Yes	54/70	(77)

* Mother or father of Melanesian origin.

**Figure 2:**
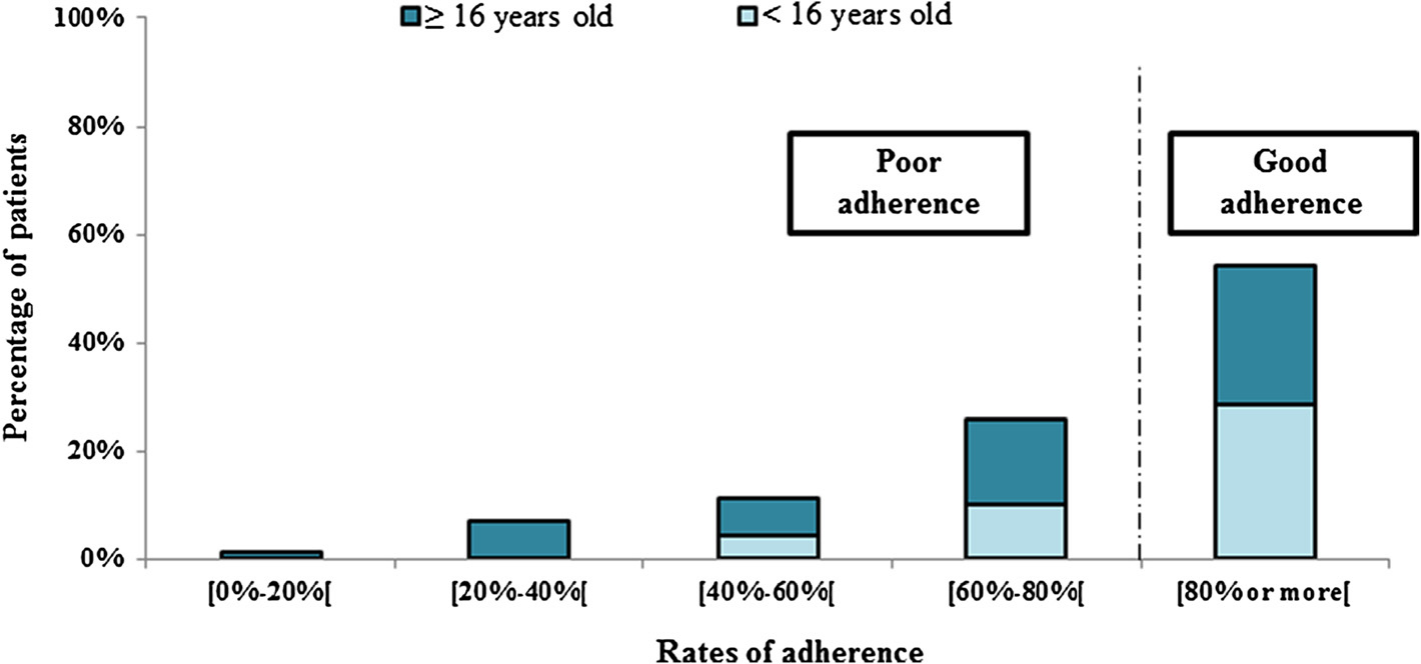
Rates of adherence in patients receiving antibiotic prophylaxis for acute rheumatic fever recurrence, Lifou, New Caledonia, 2011.

Our study showed that there were 38 good-adherent patients (54%) and 32 poor-adherent patients (46%) (Figure 2). The mean adherence to secondary prophylaxis was 77% ± 22 and the median adherence was 82.2% (IQR 76.5- 94.1). The median number of injections was 14 (range 2–18).

Public health centers followed up 65 patients, GPs followed up the remaining five. The mean distance between the house and the nearest health center was 12 km ±10.8. A fifth (21%) of the patients was administered injections at home. A total of 57 patients had a history of ARF, including 3 cases of chorea. Among the 54 patients with a previous history of RHD, 8 were diagnosed during an echographic screening. The mean duration of treatment was 6.4 ±4.9 years. The majority of people trusted the treatment (94%) and didn’t want to switch for an oral antibiotic prophylaxis (85%). Results of the univariate analysis comparing good-adherent and poor-adherent are presented in Table 2.

**Table 2:** Univariate analysis comparing good-adherent to poor-adherent patients, Lifou, New Caledonia, 2011

**Characteristics**	**Good-adherent patientsn/N (%)**	**Poor-adherent patientsn/N (%)**	P value^*^
			
**Demographic and socio-economic characteristics**			
Age <16 years old			0.092
No	18/38 (47)	22/32 (69)	
Yes	20/38 (53)	10/32 (31)	
Male sex			0.215
No	21/38 (55)	23/32 (72)	
Yes	17/38 (45)	9/32 (28)	
Concrete dwelling			0.466
No	25/38 (66)	18/32 (56)	
Yes	13/38 (34)	14/32 (44)	
Number of persons in the household ≥ 6			0.054
No	13/38 (24)	19/32 (59)	
Yes	25/38 (66)	13/32 (41)	
Other case(s) of ARF in the household			0.320
No	30/37 (81)	29/32 (91)	
Yes	7/37 (19)	3/32 (9)	
Household income <150 000 CFP (1,600 USD)/month			1
No	14/38 (37)	10/29 (34)	
Yes	24/38 (63)	19/29 (66)	
**Health care team and system-related characteristics**			
Follow up of ARF/RHD in a health center			1
No	3/38 (8)	2/32 (6)	
Yes	35/38 (92)	30/32 (94)	
Follow up of ARF/RHD in the health center of Xépénéhé			0.140
No	13/35 (37)	17/30 (57)	
Yes	22/35 (63)	13/30 (43)	
Distance between the residence and the nearest health center >5 km			0.116
No	14/38 (37)	6/32 (19)	
Yes	24/37 (63)	26/32 (81)	
Duration for an injection >3 h ^§^			0.257
No	23/29 (79)	24/26 (92)	
Yes	6/29 (21)	2/26 (8)	
BPG injections’ recall system ^¶^			0.042
No	37/37 (100)	28/32 (88)	
Yes	0/37 (0)	4/32 (12)	
Adequate healthcare coverage			0.070
No	8/37 (22)	14/32 (44)	
Yes	29/37 (78)	18/32 (56)	
**Condition-related characteristics**			
Previous history of ARF			0.121
No	10/38 (26)	3/32 (9)	
Yes	28/38 (74)	29/32 (91)	
Previous history of RHD			1
No	9/38 (24)	7/32 (22)	
Yes	29/38 (76)	25/32 (78)	
Severe RHD at diagnosis^**^			1
No	25/29 (86)	19/22 (86)	
Yes	4/29 (14)	3/22 (14)	
Hospitalization at diagnosis of ARF or RHD			0.813
No	18/36 (50)	17/32 (53)	
Yes	18/36 (50)	15/32 (47)	
**Therapy-related characteristics**			
Treatment duration >2 years			0.743
No	5/37 (14)	6/32 (19)	
Yes	32/37 (86)	26/32 (81)	
Severe pain from BPG injection^¶^			0.130
No	36/38 (95)	26/32 (81)	
Yes	2/38 (5)	6/32 (19)	
Desire for oral treatment^¶^			1
No	32/38 (84)	24/28 (86)	
Yes	6/38 (16)	4/28 (14)	
Confidence in treatment^¶^			0.316
No	1/36 (3)	3/29 (10)	
Yes	35/36 (97)	26/29 (90)	
**Patient-related characteristics**			
Protestant religion			0.602
No	10/38 (26)	11/32 (34)	
Yes	28/38 (74)	21/32 (66)	
Knowledge of treatment’s objectives			1
No	2/32 (6)	2/27 (7)	
Yes	30/32 (94)	25/27 (93)	

CI denotes confidence interval, ARF denotes acute rheumatic fever and RHD denotes rheumatic heart disease.

*P values were calculated with the use of Fisher’s exact test for categorical variables and Student’s test for continue variables.

§ The duration includes transport from home to health center, way and back. Patients having injections at home were excluded.

¶ Declarative information.

** The classification of severity was obtained by echocardiography medical records.

### Multivariate analysis

The logistic non-conditional multivariate analysis identified three independent protective factors of poor adherence: a household with more than five people (OR, 0.25; 95% CI, 0.08 to 0.75), a previous medical history of symptomatic ARF (OR, 0.20; 95% CI, 0.04 to 0.98) and an adequate healthcare coverage (OR, 0.21; 95% CI 0.06 to 0.72) (Table 3).

**Table 3:** Determinants of poor adherence to antibiotic prophylaxis (multivariate analysis), Lifou, New Caledonia, 2011

**Variables**	**Odds Ratio (95% CI)**	P Value*
Number of persons in the household ≥6		0.014
No	1	
Yes	0.25 (0.08 – 0.75)	
Previous history of ARF		0.047
No	1	
Yes	0.20 (0.04 – 0.98)	
Adequate healthcare coverage		0.013
No	1	
Yes	0.21 (0.06 – 0.72)	

CI denotes confidence interval, ARF denotes acute rheumatic fever.

*p values were calculated with the use of logistic-regression analysis.

Variables candidates were : age <16 years old, male sex, distance from nearest health center >5 km, BPG injections’ recall system, follow up of ARF/RHD in health center of Xépénéhé, severe pain from BPG injection, number of persons in the household ≥6, previous history of ARF, full medical coverage.

p value was 0.67 by the Hosmer–Lemeshow test for goodness of fit.

## Discussion

To our knowledge, this is the first study evaluating rates and determinants of adherence to secondary antibiotic prophylaxis for ARF recurrence in New Caledonia. Our study showed that there were 38 good-adherent patients (54%) and 32 poor-adherent patients (46%) in Lifou. This means that 46% of these patients with a previous history of ARF or RHD and receiving BPG injections were at high risk of recurrence of ARF.

The mean and median rate of adherence we found (77% and 82.2% respectively) was lower than the one observed in India par Kumar *et al.* (mean adherence >90%), but was higher than those assessed in Australia by Stewart *et al*. (median rate of injections 54%), by Eissa *et al.* in a remote Top End Aboriginal community (58% of patients received <80% of doses), by Mincham *et al.* in the Kimberley region in Western Australia (median adherence = 67%) and by Seckeler *et al.* in Northern Mariana Islands (median adherence = 69.2%) [16,18,19,21,25]. A study conducted in Egypt showed that 64.6% of patients had an adherence ≥84%, nevertheless the study samples can hardly be compared (20% of children under oral antibioprophylaxis, injections of BPG delivered every 2 weeks) [17]. Ehmke *et al.*'s study in Iowa, where the overall adherence was 64.6%, presents the same objections to comparison: paediatric population, oral antibioprophylaxis [26].

The wide range of indicators used in these different studies makes comparison particularly difficult and highlights the necessity of standardized indicators to evaluate adherence.

In our study, the multivariate analysis could determine three independent factors protectors of poor adherence: a demographic factor, ≥6 individuals in the household; a health system-related factor, an adequate healthcare coverage; a condition-related factor, a previous history of symptomatic ARF. Two studies, conducted in the USA, tried to determine an association between the number of individuals of the household and the adherence. Gordis *et al.* in 1969, and Ehmke et al. in 1980 found that a large number of siblings was a risk factor of poor adherence [26,27]. In our study, we postulate that a large number of siblings in the household could improve the adherence probably because of the presence of a big brother or sister. They could accompany the child to the health center or take care of the household during the time of the consultation. This hypothesis is consistent with Gordis e*t al.* study in which being unaccompanied by parent at clinical visits was associated with poor adherence. Mincham and Harrington, in two qualitative studies conducted in Australia, showed that an active recall system was a major determinant of adherence [28,29]. This secondary prevention strategy is indeed highly recommended by the WHO and the World Heart Federation [4,6].

The association between the cost of medication and a poor adherence affects particularly low income populations [15,30-32]. To our knowledge no study ever analyzed this association in secondary prophylaxis to ARF and RHD, probably because it is usually free of charge in most countries. In Lifou, BPG injections are free but the copayment required for cardiologist reviews and transports to the cardiologist might have led to a negative perception of the health system and a feeling of not “belonging” to the health service. According to Harrington and Mincham, those representations of health system were responsible of poor adherence among Aboriginal patients [28,29]. An adequate healthcare coverage might be the consequence of a good adherence. Indeed, good adherent patients having frequent interactions with health staff could have a better track of their administrative and medical record.

Patients with a previous history of symptomatic ARF had a better adherence than patients who were diagnosed with RHD without history of ARF. It is described that among chronic diseases, asymptomatic diseases (diabetes, hypertension, osteoporosis…) are more likely to generate poor adherence [15]. Patient’s perception of the disease gets modified by the existence of symptoms and the level of disability. Gordis *et al.* determined that having ARF with “no restriction of activity” was a risk factor of poor adherence among children [33]. Fear of recurrence of intense and painful symptoms might enhance adherence, unlike those diagnosed with RHD without history of ARF. Health education and sensitization in order to modify risks perception shall therefore be promoted.

Our retrospective cohort study of patients receiving antibiotic prophylaxis for ARF recurrence in Lifou was quite exhaustive; three patients only could not be contacted. A single investigator (BG) interviewed patients and collected data, therefore limiting the risk of variability. The study was led on a small island where all the health structures could be visited, which facilitated the data collection. By screening data, this study allowed us to register three new patients in the local register, to realize numerous demands for full medical coverage, to update medical and personal data and to provide information to cease two patients’ antibiotic prophylaxis. Nevertheless, one of the limitations of this study, beside its retrospective design, was the sample size, resulting in a lack of statistical power. Another limitation was that we were not able to perform a sub-group analysis comparing children aged less than16 years and adults because of the small sample size giving a lack of power.

## Conclusions

To improve adherence to secondary prophylaxis in Lifou, we therefore propose the following recommendations arising from the results of this study: i) identifying patients receiving antibiotic prophylaxis without medical history of ARF to strengthen their therapeutic education and ii) improving the medical coverage in patients with ARF or RHD. Moreover, regarding the difficulty to identify ARF/RHD patients and to collect data on the dates of injections during the study, we also recommend that the nurse designated for the ARF prevention program in Lifou coordinate an active recall system based on an updated local register. Further epidemiologic research to better answer the question as to what makes some patients adhere to their injection and others not are needed. This might include a study on the main island of New Caledonia with a bigger sample size and a randomized study evaluating an intervention. But the key point to improve adherence among Melanesian patients is probably to give appropriate information regarding the disease and the treatment, taking into account the Melanesian perceptions of the disease.

## Abbreviations

ARF: Acute rheumatic fever; RHD: Rheumatic heart disease; GP: General practitioner; WHO: World Health Organization.

## Competing interests

Bernard Rouchon is the director of the Agence Sanitaire et Sociale which leads the rheumatic fever prevention program in New Caledonia.

## Authors’ contributions

BG and EDO conceived the study and questionnaire, carried out the data collection, interpreted the data, and drafted the manuscript. EDO did the analysis. NB participated in the design of the study and questionnaire and helped to draft the manuscript. BR participated in the design of the study and questionnaire and in the data collection. JMM participated in the data collection and revised the manuscript. IdF participated in the data collection and revised the manuscript. All authors participated, read and approved the final manuscript.

## Pre-publication history

The pre-publication history for this paper can be accessed here:

http://www.biomedcentral.com/1471-2458/13/131/prepub
